# Integrated Microfluidic Membrane Transistor Utilizing Chemical Information for On-Chip Flow Control

**DOI:** 10.1371/journal.pone.0161024

**Published:** 2016-08-29

**Authors:** Philipp Frank, Joerg Schreiter, Sebastian Haefner, Georgi Paschew, Andreas Voigt, Andreas Richter

**Affiliations:** 1 Polymeric Microsystems, Institute for Semiconductors and Microsystems, Technische Universität Dresden, Dresden, Germany; 2 Highly-Parallel VLSI-Systems and Neuro-Microelectronics Chair, Institute for the Fundamentals of Electroncis, Technische Universität Dresden, Dresden, Germany; 3 Center for Advancing Electronics Dresden (cfaed), Dresden, Germany; Texas A&M University College Station, UNITED STATES

## Abstract

Microfluidics is a great enabling technology for biology, biotechnology, chemistry and general life sciences. Despite many promising predictions of its progress, microfluidics has not reached its full potential yet. To unleash this potential, we propose the use of intrinsically active hydrogels, which work as sensors and actuators at the same time, in microfluidic channel networks. These materials transfer a chemical input signal such as a substance concentration into a mechanical output. This way chemical information is processed and analyzed on the spot without the need for an external control unit. Inspired by the development electronics, our approach focuses on the development of single transistor-like components, which have the potential to be used in an integrated circuit technology. Here, we present membrane isolated chemical volume phase transition transistor (MIS-CVPT). The device is characterized in terms of the flow rate from source to drain, depending on the chemical concentration in the control channel, the source-drain pressure drop and the operating temperature.

## Introduction

Lab-on-a-chip-technology as an integrated circuit (IC) concept is intended to provide economical and functional advantages in microfluidics in a manner comparable to microelectronics in electronic information technology. However, Albert Folch noted that “…this vision is not as fully realized as the exponential progression of integrated circuit improvement known as Moore’s law, simply because there is no such thing as a microfluidic transistor on which to sustain the same economy of scale as there was for the electronic transistor.” [1].

The dominating microelectromechanical system (MEMS)-based lab on a chip (LoC) technology employs computer controlled microfluidic components to carry out microfluidic IC programs [2]. Whereas electronic ICs are the heart of computers, current MEMS-based LoCs are simply complex computer-controlled devices. One common approach is the use of elastomeric valves that can be activated by a pneumatic or hydraulic input signal, and output a pre-determined microfluidic signal. Using this concept, basic circuits like logic gates and oscillators have been shown [3–6] constituting fluid-mechanical counterparts to the electronic circuits.

Against this background, there is a high motivation to investigate the possibilities of a microfluidic transistor concept as the component base for integrated circuits, eventually providing a comparable economy of scale in microfluidics as there is for microelectronics.

While in microelectronics electric charges transport information, in microfluidics the fluid containing reactive molecules and ions is the carrier of information. This extends the scope of information channels and information types of the technology resulting in not only the manipulation of information, but of matter. At the core of our concept, a microfluidic, (or better: chemo-fluidic) transistor connects the signal domain of the fluid as multi-dimensional carrier of information with the mechanical or microfluidic domain. The coupling of domains is facilitated by intrinsically active materials such as stimuli-responsive hydrogels, which react to small chemical changes in a fluid with a volume phase transition, where the gels undergo a significant change in volume.

Typical devices facilitating valving in micro channels such as pneumatic membrane valves [7], vacuum valves [8], thermally [9] or mechanically activated valves [10] rely on externally applied energy in order to function. The energy is triggered via a control signal, which also originates from an external control setup *e.g.* a computer with according circuitry. The chemo-fluidic membrane transistor in contrast uses the input from the process liquid in order to make a switching decision, even more so it is able to conduct a fluid with according fluids to subsequent chemo-fluidic transistors, and therefore carry out a signal propagation. Most microfluidic valves conceptionally lack the ability to effect the switching decision of the subsequently following device, contradicting an autonomous information processing and further a conclusive IC concept.

Inspired by microelectronics, the chemo-fluidic transistor paves the way for a transistor-based circuitry as an on-chip flow control extracting inputs directly from the process fluid, eventually rendering all external control devices obsolete. In our concept, integrated circuits, just like in electronics, conduct the manipulation (of fluid) purely on the chip level. In order to lay the foundation for this comprehensive and complex circuit concept for LoCs we describe in this article the key device, the chemo-fluidic transistor. The transistor offers a flow control in one channel while the signal input is given by a separate channel. The channels are coupled hydraulically via a flexible membrane instituting the devices name: membrane-isolated chemo-fluidic volume phase transition transistor (MIS-CVPT), or chemo-fluidic membrane transistor.

### Hydrogels as active material

Similar to the electronic model, the MIS-CVPT is a monolithic device, whose functioning relies on intrinsically active materials, here stimuli-responsive hydrogels. These polymers show unique material properties. Hydrogels are three-dimensional networks of polymer chains synthesized via a cross-linking polymerization or a cross-linking reaction [11]. The polymers building hydrogels show solubility in water. While a polymer will dissolve in its solvent, a cross-linked polymer, due to its interconnections, will not dissolve but retain the solvent within its network, leading to a swelling of the structure. The equilibrium swelling degree depends on the solubility in the solution utilized.

Further, stimuli-responsive hydrogels exhibit a reversible first order volume phase transition. By small changes of the thermodynamic properties of the solution, the gel can be made to undergo a volume phase transition between a shrunken state and a swollen state. In the shrunken state the polymer-polymer interactions are prevalent and the gel is highly hydrophobic. In the swollen state (mixing state) solvent-polymer interactions are dominant. Hydrogels have been designed to show sensitivity to a large variety of thermodynamic parameters including concentrations of solvents, specific ions, biomolecules and pH value [12–17], but also to electrical field [18], light [19] or temperature [20].

For the MIS-CVPT a co-polymer of poly(*N*-isopropylacrylamide) (PNIPAAm) and the superabsorbent sodium acrylate (NaA) is employed. The composition of both monomers combines the sensitivities of NIPAAm (alcohol and temperature sensitivity [21, 22]) with the high swelling degree of NaA. The swelling behavior of PNIPAAm and the PNIPAAm-NaA composition is shown in S1 Fig over the ethanol concentration. The co-polymer exhibits a higher swelling degree, while showing a change of equilibrium volume at roughly the same values of ethanol concentration as pure PNIPAAm. The gel particle is produced photolithographically [23] and is placed in the device acting mechanically on chemical inputs by changing its volume.

### Device principle

**Fig 1:**
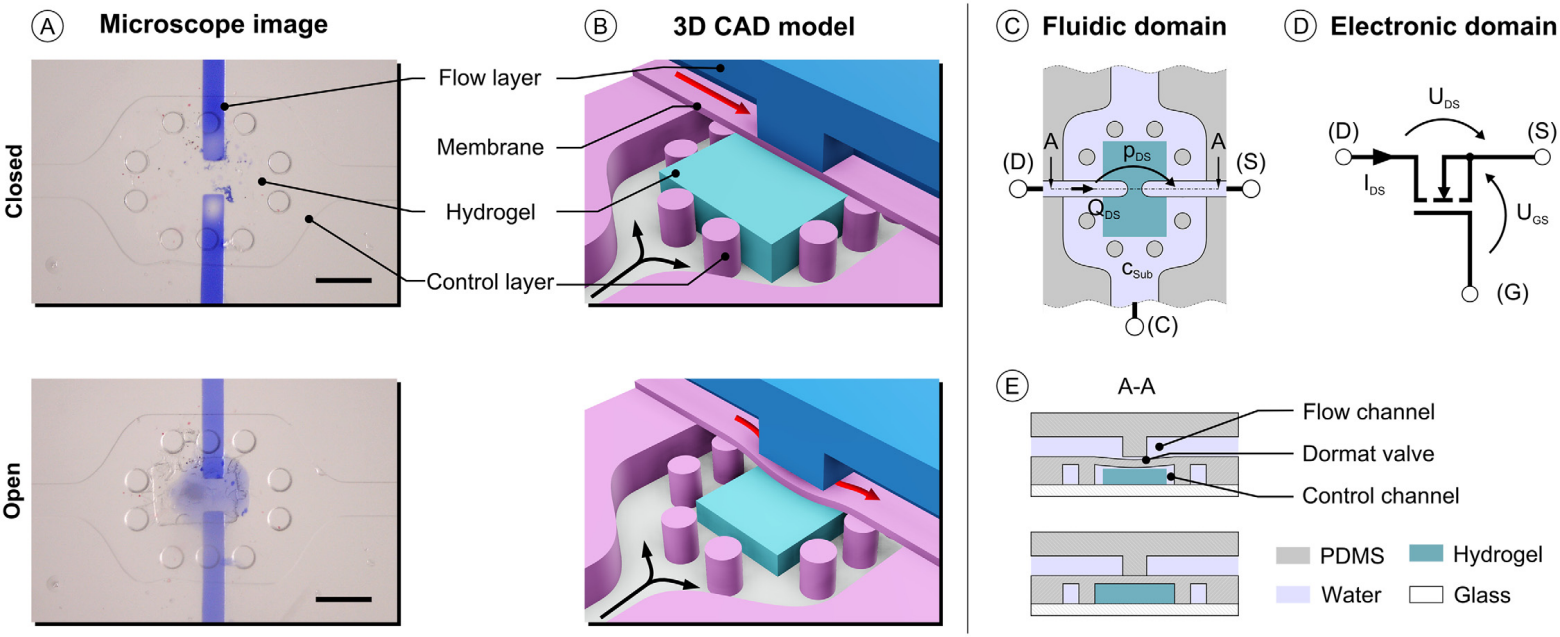
(A), Microscope image of the MIS-CVPT (scale bar 500 μm). (A-top), Fully swollen hydrogel closing the transistor for a low substance concentration. (A-bottom), Shrunken hydrogel opening the transistor for a high substance concentration. (B), Schematic 3D CAD model of the MIS-CVPT closed and open. (C-D), Comparison of fluidic and electronic domain, devices with according terminals. Voltage corresponds to pressure and current corresponds to flow. (C), Schematic top view of the MIS-CVPT with analogue electronic ports of the MOSFET. (D), Graphical symbol of the n-type enhancement MOSFET with its terminals. (E), Cross section view with open and closed state of the MIS-CVPT including the used materials.

The chemo-fluidic membrane transistor consists of two operational layers, the flow layer and the control layer including a hydrogel particle, which are separated by a thin flexible membrane (Fig 1A–1C and 1E) derived from a series of works [24–27] but specifically extending the concept of the chemo-mechanical valve [21, 28]. The thermal responsiveness of hydrogels as microfluidic valve has been exploited before by [9], but without utilizing the coupling of chemical and fluidic domain. The flow layer is interrupted by a channel break, forming, in combination with the membrane, a doormat-like valve [29–31]. The doormat setup was chosen as it closes a channel tightly, in contrast to a membrane valve setup without the channel break. In order to ensure good tightness of the valve the flow channel was designed to be smaller than the underlying hydrogel particle. A microscope picture of the device is given in Fig 1A for the closed and open state. Typically in the closed state, where the hydrogel is fully swollen, the gel particle is not visible, because its refractive index is close to the one of water, making it indistinguishable from the aqueous phase.

In reference to the electric-fluidic analogy, the MIS-CVPT is presumed to be the counterpart of the n-type enhancement metal-oxide-semiconductor field-effect transistor (MOSFET) in electronics: Drain-source voltage U_DS_ corresponds to the excitation pressure p_DS_ from (D) to (S) in the flow channel (Fig 1C and 1D), the drain-source current I_DS_ corresponds to the flow rate Q_DS_ in the flow channel. Gate-source voltage U_GS_, which is the control voltage, corresponds to the substance concentration c_sub_ (port (C)), which determines the pressure applied by the hydrogel against the membrane (Fig 1E). At c_sub_ = 0wt% the pressure applied by the gel is high and decreases with increasing substance concentration. Analogue to the n-type enhancement MOSFET the MIS-CVPT is also normally non-conductive. Similar to the threshold voltage of a MOSFET, there is a minimum concentration c_sub,0_ for the MIS-CVPT to open. While in electronics temperature is often regarded a perturbation, for the chemo-fluidic transistor it is more of an additional control input, and can be used e.g. to adjust the threshold. In the MOSFET, the gate is electrically isolated from the source-drain channel. Likewise, there is no fluid flow from the control port to the source-drain channel in the MIS-CVPT. The sensitivity and specificity to a certain input is bound directly to the composition of the gel. By changing the composition of the gel a different information can be processed and be utilized as trigger. PNIPAAm for instance is known for a distinct sensitivity towards a series of alcohols [21] and towards temperature change [32]. For this study we use a copolymer of PNIPAAm and sodium acrylate. This gel composition has shown to have a high swelling degree from the sodium acrylate while still keeping the sensitivity of the PNIPAAm [33]. The concentration c_Eth_ of ethanol is used as the chemical input of the chemo-fluidic membrane transistor.

## Experimental

**Fig 2:**
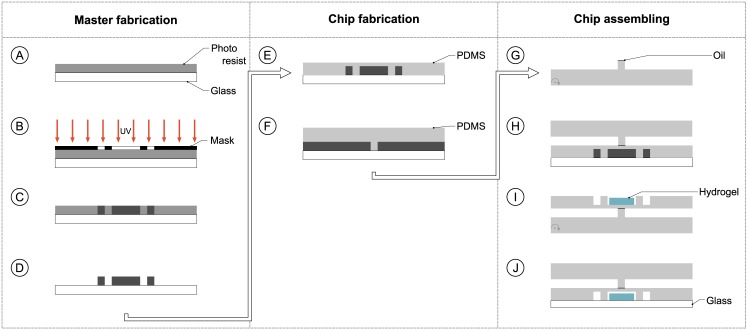
Overview of the fabrication procedure. **Master fabrication:** (A) Lamination of dry film resist onto substrate. (B) Exposure with UV light through photo mask. (C) Post-exposure bake. (D) Development and rinsing with following hard bake. **Chip fabrication**: (E) Spin coating of PDMS on control layer master. (F) Moulding of PDMS on flow layer master. **Chip assembling**: (G) Inhibition of the channel break in the flow layer. (H) Plasma bonding with aligning of the PDMS layers. (I) Incorporation of the hydrogel particle into the control channel. (J) Plasma bonding of the multi-layer chip onto a cover glass.

The chemo-fluidic membrane transistor was fabricated using multi-layer soft lithography (Fig 2). Prior to the PDMS moulding process photolithographically structured masters for each functional layer were produced. While the control layer was produced by spin coating to achieve a thin layer the flow layer was casted resulting in a fairly thick layer. The assembling of the layers and the incorporation of the hydrogel particle concluded the fabrication process.

### Master fabrication

An overview about the master fabrication is given in Fig 2A–2D. A glass substrate (Borofloat33, Schott Jena, Germany) with a thickness of 700μm was cleaned with acetone, isopropanol as well as distilled water and consecutively dried using nitrogen. A dehydration bake at 150°C for 20 min was performed on a hot plate as all following baking steps. One layer resist (Ordyl SY355, Elga Europe, Italy) of thickness 50μm was laminated and soft baked at 85°C for 3 min. This step was repeated for a second layer of 50μm (Fig 2A). The substrate was exposed through a polymer film mask for 90 s for the flow layer and 65 s for the control layer (Fig 2B). The intensity was previously measured and determined to be 1.66W cm^−2^. The post-exposure bake was performed at 85°C for 30 min (Fig 2C). For development the Ordyl developer and rinser (Ordyl Developer and Rinser, Elga Europe, Italy) was used. The development was finished off with rinsing with isopropanol and distilled water (Fig 2D). The process was completed with a hard bake at 120°C for 1.5 h.

### Chip fabrication

#### Control layer

The control layer contains the membrane which has a thickness of about 30 μm. Therefore spin-coating was chosen as the method of fabrication. 22.0 g of PDMS (Sylgard 184, Dow Corning, USA) in a ratio 10:1 is mixed with an electric stirrer for 5 min. It is then degased for 45 min. The bubble-free PDMS was used to spin coat the control master (Fig 2E) creating a layer thickness of 130 μm. The PDMS was then cured in a convection oven at 60°C for 2 h.

#### Flow layer

27.5 g of polydimethylsiloxane (PDMS) (Sylgard 184, Dow Corning, USA) in a ratio 10:1 was mixed with an electric stirrer for 5 min. It was then degased for 45 min. The bubble-free PDMS was poured on top of the flow master (Fig 2F) creating a layer thickness of 4 mm. The PDMS was then cured in a convection oven at 60°C for 2 h.

### Hydrogel particle

The hydrogel solution was prepared under protective argon atmosphere. For a batch of 25 ml 2.069 g PNIPAAm (poly(*N*-isopropylacrylamide), Sigma-Aldrich, USA), 0.044 g Sodium acrylate (Sigma-Aldrich, USA), 0.043 g N,N’-Methylenbisacrylamide (Sigma-Aldrich, USA) and 0.042 g photo initiator (2-methylpropiophenone, Sigma-Aldrich, USA) were weighed out. The solids were dissolved in 25 ml de-ionized water. The hydrogel solution was pipetted into a PDMS mould under argon protective gas and closed up with a glass slide. The mould was then exposed to UV light for 20 s. The gel structures were dried in the mould at room temperature overnight.

### Chip assembling

The assembling is additionally documented in Fig 2G–2J. Prior to the bonding of the two functional layers, the doormat setup in the flow layer is inhibited from bonding by dispensing a small drop of oil on top (Fig 2G). The prepared flow layer was placed into a plasma oven (DREVA Clean 450, Vakuumtechnik Dresden GmbH, Germany) together with the control layer (still on the master). The two layers were exposed to an oxygen plasma for 2 min at 50W with an oxygen flow of 20 sccm. After treatment, the two layers were aligned (Fig 2H). The fluidic connections were punched with a biopsy tool, diameter of 1.2 mm. The hydrogel structure was then placed in the chamber in the control layer (Fig 2I) and the chip was bonded on top of a glass substrate (Fig 2J) via oxygen plasma activation (as above).

### Measuring setup

The flow channel inlet was supplied with pressure by a pressure flow pump (AF-1, Elveflow, France), while the outlet was set to ambient pressure. Distilled water was used as the fluid. The flow rate was measured with a flow sensor providing a flow range from 0 μL min^−1^ to 50μL min^−1^ (Flow sensor 50 μL min^−1^, Elveflow, France). The control channel was supplied with a syringe pump (LA-100, HLL Landgraf Laborsysteme, Germany) at a constant flow of 15 μL min^−1^. For perfusing the control channel incorporating the hydrogel a solution of ethanol at various concentrations diluted in distilled water was used. The microfluidic chip system was placed on a tempering element connected to a circulation thermostat (Haake A10, Thermo Fischer Scientific Inc., USA) at a constant temperature for each particular experiment. The temperature compliance of the thermostat was regularly checked with a digital thermometer (Qtemp 600, VWR International GmbH). The chip was characterized in a stationary and dynamic manner, the dependent variable was always the flow rate. For the stationary characterization ethanol concentration, temperature and pressure were varied. The ethanol concentration the gel was exposed to was varied in a range from 0 wt% to 15.0 wt% in 2.5 wt% steps. The temperature was varied in a range from 10.0°C to 30.0°C in 2.5K steps. A home-made Python software was employed for controlling the pressure pump. The inlet pressure of the flow channel was varied in a range from 0mbar to 700mbar in 25mbar steps. During the measurement the pressure was applied for 5 s and then released for 15 s, this was repeated three times for each pressure step. For the dynamic characterization one operating point of ethanol concentration (c_*Eth*_ = 2.5wt%) and temperature (*ϑ* = 20.0°C) was chosen, and a sine pressure function was applied. The period of the sine function was varied, the amplitude of the sine was 50mbar, and the offset wa 400mbar. The measured data was analyzed and processed with home-made Python software (Python 2.7). In the dynamic investigation as pressure driven flow pump we used the system described in [34] in connection with a flow sensor (SLI-1000, Sensirion AG, Switzerland).

## Results and discussion

As the chemo-fluidic membrane transistor is a novel microfluidic device, it is important to analyze its behavior to draw conclusions for its future applicability. Here we present and discuss a quantitative characterization of the device when controlled by a static source-drain-pressure (“output characteristic”) and a static chemical control concentration (“transfer characteristic”). In addition we show the dynamic reaction both to a change in pressure and in chemical concentration. We present a quasi-static model of the MIS-CVPT to be used for circuit design, and finally discuss the potential of the device within the field of microfluidics.

### Output characteristic

**Fig 3:**
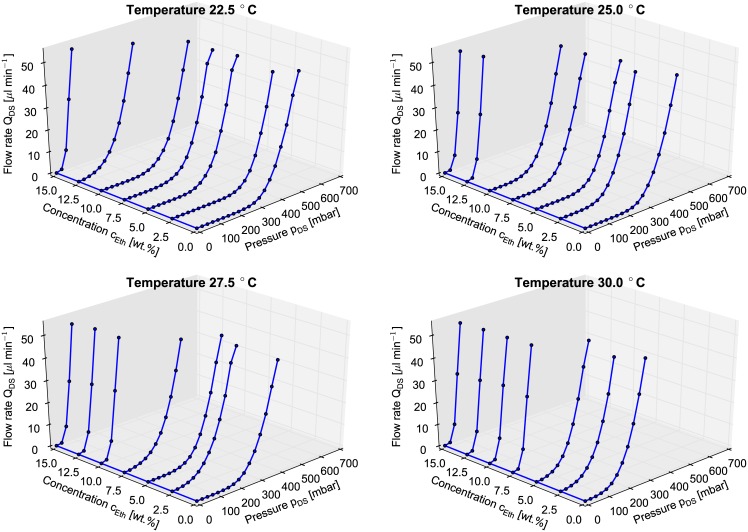
3D-plot of measured output characteristics (flow rate Q_DS_ vs. pressure p_DS_ vs. ethanol concentration c_Eth_) of the MIS-CVPT at different temperatures *ϑ* ranging from 22.5°C to 30.0°C in 2.5K steps.

**Fig 4:**
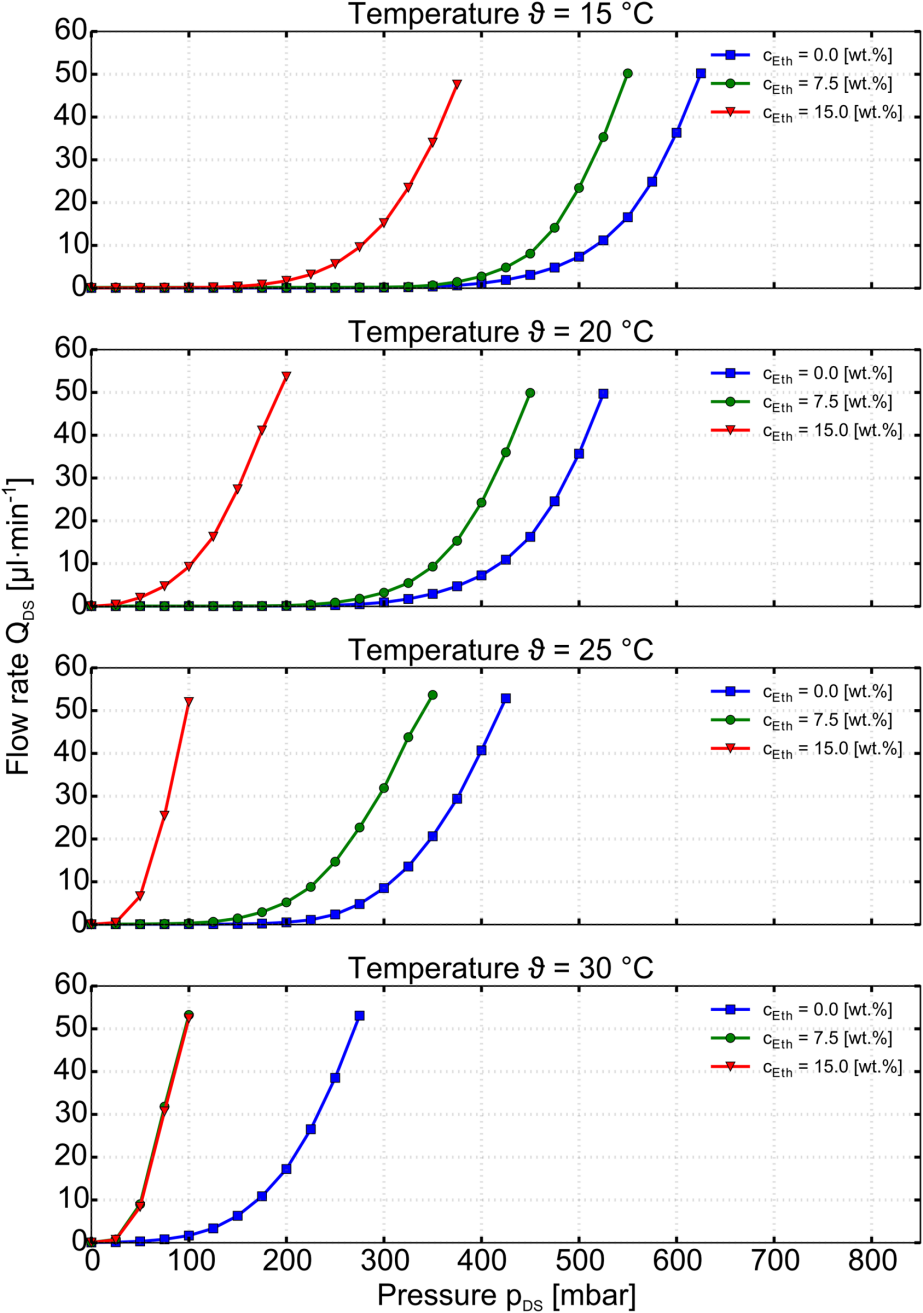
Measured output characteristics (flow rate Q_DS_ vs. pressure p_DS_) of the MIS-CVPT with the different ethanol concentrations c_*Eth*_ 0wt%, 7.5wt%, and 15.0wt% at different temperatures *ϑ* ranging from 15.0°C to 30.0°C in 5.0K steps.

Figs 3 and 4 show the output characteristic of the chemo-fluidic membrane transistor in terms of the flow rate Q_DS_ versus the excitation pressure p_DS_. In order to give an overview, Fig 4 shows the data over the entirety of the parameter field in four 3D-plots.

The drain-source channel of the MIS-CVPT becomes conductive for pressures higher than the opening pressure which depends on both temperature and ethanol concentration. This opening pressure decreases with increasing temperature and increasing ethanol concentration. Fig 4 shows a selection of fewer data for better clarity. Here the graphs of the MIS-CVPT for ethanol concentration 0wt%, 7.5wt% and 15.0wt% over the temperatures *ϑ* from 15°C to 30°C are plotted in 5.0°C steps. The increase of temperature as well as the increase in concentration causes the gel to shrink, releasing the pressure off the membrane. In case of the curve for 0wt% ethanol concentration the opening pressure decreases T from 425mbar down to 100 mbar by a change of temperature of 15K. The curve for 15.0wt% ethanol concentration at 25°C remains the same for 30.0°C.This behavior can be explained by the fact that the gel is collapsed to a degree where it does not influence the switching of the membrane anymore. So for this part the mechanical properties of the doormat setup dominate the behavior of the chemical transistor completely, *i.e.* no chemical input is handled anymore, only the passive flow resistance remains. The device is in a state of overdrive, where an additional increase of concentration will not change the behavior. The curve for 7.5wt% at 30.0°C shows the same behavior. For the hydrogel a saturation of the ethanol concentration sets in. For every temperature step of 2.5K the next higher ethanol concentration in 2.5wt% steps reaches a saturation for the gel particle to shrink. When the device is in the overdriven state, the graphs of flow rate vs. p_DS_ are the same, independent of temperature and concentration. In order to assure that the pressure drop in the control channel dos not influence the opening behaviour, the flow was limited to 15 μl min^−1^. We simulated the according pressure in an CFD simulation. The results presented in S2 Fig and S1 File show a neglectable magnitude of the pressure.

At higher pressures, the output characteristic of the MIS-CVPT is expected to reach saturation with only linear increase of the flow rate. According to the design of the device, the opening between membrane and channel break has a limited size. This would lead to a constant conductance of the channel and hence a linear dependence of flow rate vs. pressure. In the current set-up the device characteristic could not yet be obtained for high values of p_DS_, since the flow sensor saturates at 50μL min^−1^. The expected saturation however would correspond to the saturation region in the output characteristic of the electronic MOSFET.

### Transfer characteristic

**Fig 5:**
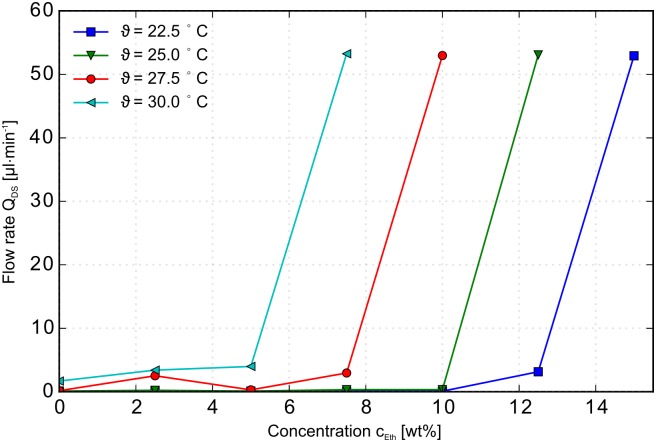
Transfer characteristic of the MIS-CVPT. Flow rate Q_DS_ over ethanol concentration c_Eth_ at a constant pressure p_DS_ = 100mbar for the temperatures *ϑ* 22.5°C; 25.0°C; 27.5°C; 30.0°C.

The transfer characteristic of a MOSFET describes the relation of Drain-Source current I_DS_ versus the Gate-Source voltage U_GS_. In the case of the MIS-CVPT this relation is described by the flow rate Q_DS_ versus the ethanol concentration c_Eth_. The corresponding graphs for the transfer characteristic of the MIS-CVPT are shown in Fig 5 for several temperatures. The graphs are at constant pressure 100mbar p_DS_. The concentration determines the flow rate through the device. With rising ethanol concentration the MIS-CVPT becomes conductive. The temperature in this model can be used for biasing of the MIS-CVPT and hence corresponds to the bulk voltage U_b_ in a MOSFET. Fig 5 demonstrates, that the concentration at which the MIS-CVPT becomes conductive (threshold concentration) can be adjusted via the change of the temperature *ϑ*. Here an increase of 2.5K in temperature decreases the concentration at which the device opens by 2.5wt% ethanol.

### Dynamic behavior

**Fig 6:**
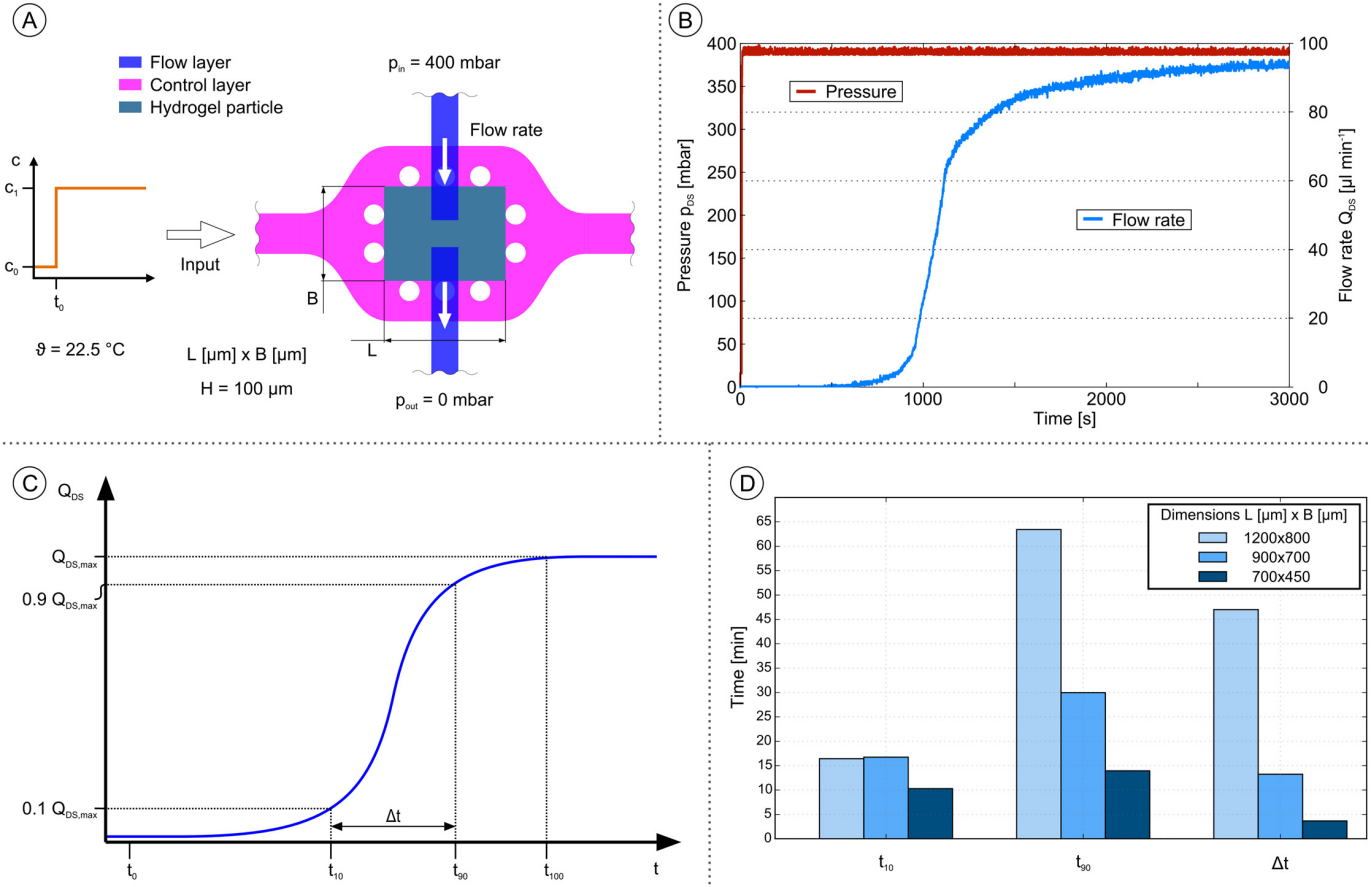
Dynamic investigation of the gate switching. (A), Experimental approach of the investigation. Switch event at t_0_ carried out by a change of the input flow in the control channel from c_0_ (= 0wt%) to c_1_ (= 30wt%), while applying a constant pressure over the chemo-fluidic transistor. The flow rate generated in the flow channel is measured via a flow sensor. (B), Typical measurement of pressure and flow rate data over time, starting from time t_0_ (= 0s). (C), Schematic graph with characteristic parameters to quantify the experimental data. (D), Bar graph of the characteristic parameter t_10_, t_90_ and Δt for three different dimensions of gel particles.

Against the background that hydrogels are perceived to work rather slowly and keeping in mind, that the switching speed determines the future application and acceptance of such a system, the dynamic behavior of the MIS-CVPT is of particular interest. Here, we analyze the dynamic response of the transistor to a change in chemical concentration. An analysis of the response to varying pressures (mechanical response) is presented in S3 Fig and S2 File. The approach of the dynamic investigation regarding a change of chemical concentration is shown in Fig 6. In the beginning of the experiment the transistor is closed. At time t_0_ (= 0 s) the ethanol concentration in the control channel is switched from c_0_ (= 0wt%) to c_1_ (= 30wt%). c_1_ was set to be 30wt% ethanol as the swelling curve of the hydrogel (S1 Fig) exhibits minimum volume at this concentration and therefore sets the transistor to overdrive. The flow channel of the transistor is fed with a constant pressure p_DS_ of 400mbar and a flow rate sensor was installed following the chip. Over time the volume phase change transition takes place and the gel particle shrinks leading to the opening of the transistor.The flow rate in the flow channel reaches saturation plateau after some time. The dynamic investigation was conducted with three different transistor sizes. An exemplary measurement of the flow rate over time is shown in Fig 6B. In order to quantify the results three characterization parameters were derived and are marked in Fig 6C. Time t_10_ corresponds to 10% of the maximum flow rate Q_DS,max_, time t_90_ corresponds to 90% of the maximum flow rate Q_DS,max_, and the time interval Δt is the difference between t_90_ and t_10_. These three parameters indicate the response time of the system when it is exposed to a jump of ethanol from a low to a high concentration.

The results for the three tested transistor sizes (defined by the gel particle size L [μm] x B [μm]) over the characterization parameter t_10_, t_90_ and Δt are shown in Fig 6D. We systematically scaled the device from dimensions (1200 x 800) μm^2^ down to dimensions of (700 x 500) μm^2^ while keeping the height of the system constant at 100 μm. For a spherical hydrogel particle of radius *r*_0_ the characteristic reaction time can be estimated via

$$\tau =\frac{s^{2}}{\pi^{2}D_{\text{coop}}},$$ (1)

where *D*_*coop*_ is the cooperative diffusion coefficient, which is typically in the order of 10^−11^m^2^s^−1^ for neutral hydrogels [11]. As liquid supply to the gel is expected to occur mostly from the sides in the current valve set-up, the half-width *B*/2 is considered to be the characteristic length determining gel deswelling. Values of the reaction time estimated with Eq 1 and a value of *D*_*coop*_ = 5 × 10^−12^m^2^s^−1^agree with the measured valve reaction times *t*_90_ within about 30%, if the radius of the gel *r*_0_ is substituted by *B*/2. Especially, the square dependence of reaction time on length scale is roughly shown in the values of *t*_90_. If the gel is in the shrunken state, a switch from high concentration *c*_1_ back to low concentration *c*_0_ will trigger gel swelling and hence the closing of the source-drain channel. Gel swelling is typically slower than gel shrinking (*e.g.* by a factor of three reported in [35]). Therefore a qualitatively similar, but accordingly slower response of the MIS-CVPT is expected for this inverse process. The diffusion-based transport of liquid in and out the gel allows a setting of the time scale by the spatial dimensions of the gel particle. For dimensions in the range of several 10 μm the time regime changes to a scale of seconds. Hence a faster response of the MIS-CVPT can be achieved simply by reducing its size. The dynamic switching of the MIS-CVPT and a MOSFET show similarities to an extent. In both cases an element is loaded (or unloaded) at a limited rate in the switching process. For the MOSFET this element is the gate electrode. Its capacitance for electric charge depends on size parameters (area of electrode, thickness of dielectric layer) and material parameters (dielectric constant). The charging rate is determined by the capacitance and the total resistance of the charging circuit, while the internal gate resistance and the gate capacitance of the MOSFET determine its maximum operating speed. For the MIS-CVPT, the element being loaded is the hydrogel particle, which is filled with or emptied of liquid. The loading rate is determined by the relaxation of the gel network which depends on geometric parameters as well as material parameters such as elastic moduli and polymer-solvent friction. Both MIS-CVPT and MOSFET exhibit a threshold in their transfer characteristic: In the MOSFET, the source-drain channel only becomes conductive, when the gate electrode has been charged above the threshold voltage of the transistor, while in the MIS-CVPT the hydrogel has to shrink below a certain threshold size to facilitate the opening of the channel at *t*_10_. Acting together, limited charging rate and threshold behavior cause a characteristic delay after the trigger signal until the source-drain channel becomes conductive in both devices.

### Compact Model

#### Quasi-static model

**Fig 7:**
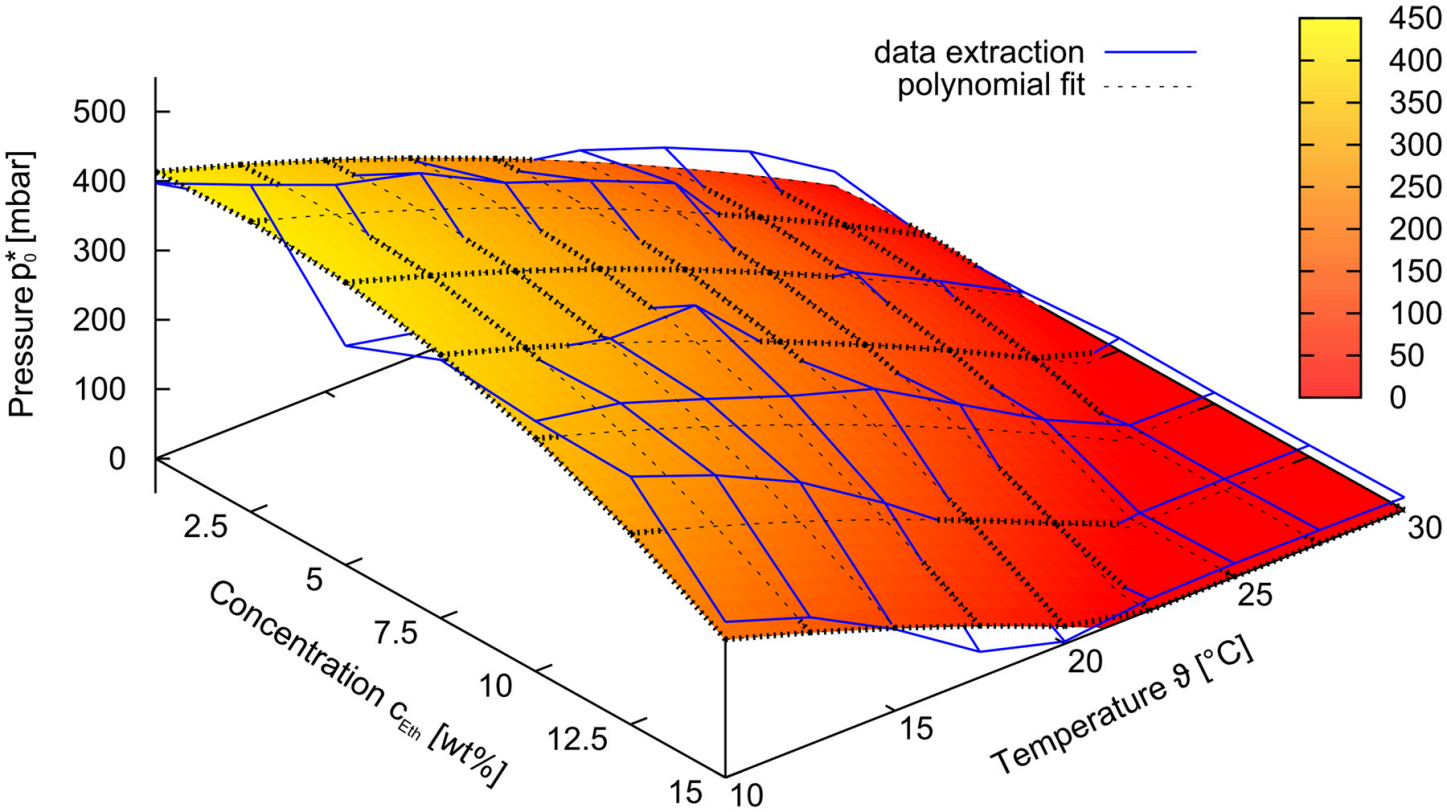
Opening pressure extracted from the measurement data and modeled as a 2nd order 2D polynomial.

In order to simulate larger circuits with design automation tools, compact models of the components involved are needed. Here we present a physically motivated quasi-static compact model of the MIS-CVPT based on a fit to experimental data. Given the temperature, the chemical concentration in the control channel, the pressure in the control channel and the source-drain pressure, the model can be used to calculate the source-drain flowrate. The model is based on assigning a fluidic conductance *G* to the source-drain channel. Analogous to Ohm’s law in electronics the flowrate from source to drain can then be calculated via *Q* = *Gp*_*DS*_, where *p*_*DS*_ is the source-drain pressure. We distinguish two cases: Either the hydrogel is swollen and always touches the membrane exerting some pressure, or the hydrogel is collapsed. We first consider the former case. If the hydrogel were allowed to swell unobstructedly, it would reach a volume *V*_*free*_. Due to mechanical constraints the gel is restricted to a size *V*_*gel*_. The pressure resulting from this compression can be roughly estimated via $K\frac{V_{free}-V_{gel}}{V_{free}}$, where *K* is the bulk modulus of the gel. If the membrane is not deflected yet (*V*_*gel*_ = *V*_*cage*_), a threshold pressure *p*_0_ needs to be overcome to open the source-drain channel for conduction. With the effective pressure *p*_*eff*_ = (*p*_*drain*_ − *p*_*source*_)/2 − *p*_*c*_ > *p*_0_ this conditition can be expressed as *p*_eff_ > *p*_0_, where *p*_*drain*_ is the pressure in the drain inlet, *p*_*source*_ is the pressure in the source outlet and *p*_*c*_ is the pressure in the control channel. For higher pressures the gel will be further compressed (with a linear dependence of volume on pressure in this approximation) and hence the membrane will be deflected further. The deflection of the membrane can lead to a deepening of the channel or a broadening of the channel. According to Hagen-Poiseuille’s law the conductivity of a wide rectangular channels shows a linear dependence on the width of the channel and a cubic dependence on the height of the channel. In the experiment, the dependence of *G* on *p*_eff_ showed to be quadratic to a good approximation, indicating a mixture of the two effects, see S4 Fig. The opening threshold pressure *p*_0_ as a function of temperature and chemical concentration was determined from the experimental data and fitted by a 2d polynomial (Fig 7).

If the gel is in the shrunken state, the membrane can be deflected by very low pressure values until it touches the gel where higher pressures will be needed for further deflection. As this exact behavior is difficult to model we decided to fit the measured data by an adequate routine instead to obtain the parameters for the model. Here, a linear dependence of the conductance on *p*_eff_ showed good agreement with experimental data, see S4 Fig.

In the current state, this model can be used to calculate steady-state phenomena by microfluidic circuit analysis tools. In future work the description of the transient behavior will be implemented as well, to allow the simulation of switching processes and oscillations. More details about the mathematical implementation of the model and a comparison of the fitted model with experimental data can be found in S3 File.

## Discussion

Both microfluidics and electronics deal with signal propagation in circuits. If we consider pure laminar flows the mathematical isomorphism is given by the hydraulic analogy, equating pressure drop with voltage and flow rate with current. Fluidic resistances can be calculated by the Hagen-Poiseuille law, capacities stem from deflectable membranes and “inductances” come from the inertia of the fluid (which is usually negligible). However, the key difference between electronics and microfluidics is the existence of (one or more) chemical concentrations within the flow. In current approaches these concentrations are usually passively transported with the flow which is controlled by purely mechanical means. While the pressure and flow rate signals travel at the speed of sound, concentration propagation is governed by transport equations. This entails flow rate-dependent signal delays. These can prove limiting in some cases, but they can also be employed for tailored stimulus/reaction control and feedback mechanisms. The similarity of electronics and microfluidics allows the employment of tools from electronic design automation, albeit in a modified form to account for chemical signals [35]. In contrast to other actuation schemes, transistors based on smart hydrogels couple from the chemical to the fluidic domain. As fluidic signals can be converted back to chemical signals by well-designed mixing-junctions, this facilitates signal transduction purely based on chemical signals. In this domain of chemical signals, circuits know from electronics, such as oscillators, comparators, logic gates and flip-flops can be built. Since no external sensing and actuation units are used, there is no limitation due to the constraints implied by external connections of the microfluidic system. Therefore hydrogel-based transistors show a high potential for large-scale integration. In addition, chemical-fluidic coupling can be utilized for the smart control of chemical quantities that are difficult or impossible to sense otherwise by external sensing units. A specific potential of the chemo-fluidic membrane transistor lies in the separation of control and flow layer, and in the use of a *channel break*. The resulting output characteristic has a well-defined region of full closure of the source-drain channel, while the *transfer characteristic* shows a sharp response to a change in concentration. This is particularly important for digital applications and sensitive species detection. In our next plans, we will build elementary circuits, wherein the device is operated at drain pressure values below the threshold pressure of the swollen state, and switching between the blocked and conducting states is controlled purely by concentration in the control channel. The *separation of control and flow layer* is the most important feature of the MIS-CVPT. In contrast to the chemo-fluidic transistor used in [35] it facilitates a much more flexible setup of chemo-fluidic control operation. The controlling flow and the controlled flow can be kept separate, can be connected, and can even be swapped in a rather arbitrary fashion by the design of the microfluidic system. In a digital setup, the separation allows for chemical signal recovery without pressure build-up, elevating the limits for cascading of logic stages. These features pave the way for complex control schemes determined by programs coded by complex chemical signals.

## Conclusion

Within this study we presented a microfluidic monolithically integrated transistor-like device that couples the chemical with the fluidic domain. The transfer and output characteristics of the device show that the chemo-fluidic membrane transistor is able to process both discrete and analogue chemical signals. Any thermodynamic parameter able to provoke a volume phase transition of stimuli-responsive hydrogels within an aqueous solutions, for example concentration of organic solvents such as alcohols, ions (*e.g.* of salts), hydrogen (pH-value) and biomolecules like glucose can be coupled with the fluidic domain via chemo-fluidic transistors. For a clearer understanding the article solely covers the characterization of the device with ethanol concentration as model system. Additionally the threshold concentration of the MIS-CVPT can be remotely controlled by the threshold temperature *ϑ*_th_.

As we aim for a rethinking of the LoC concept, we are quite aware of the importance of the dynamic behavior of such a technology in order to gain acceptance. Even though we realize the large time scale of the response, our results clearly state, that the dynamics of the device can be improved by merely scaling down the technology. By focussing our efforts on conquering the next technology node for this platform, we are confident to sustainably speed up the dynamic behavior.

As we have moved forward in our concept we have developed a series of devices contributing to the chemo-fluidic circuit concept. *E.g.* a chemo-fluidic delay line oscillator [35], which was recently published by Paschew et. al., allows the bidirectional coupling of the chemical and the fluidic domain introducing event-based control on the component level. This offers possibilities that exceed the capabilities of present-day microfluidics. The report of the chemo-fluidic oscillator also addressed the crucial importance of the utilization of computer aided design (CAD) in microfluidics. The compact model in this report follows this pave and aims for a conclusive development process incorporating CAD, modeling and simulation. Another approach demonstrated the autonomous and purely fluidic transistor-based control in contrast to the current dominant concept of computer controlled labs-on-chips. A proof of principle of complex circuit programs based on chemo-fluidic components has been shown by Greiner [36].

The next challenge that arises is the development of chemo-fluidic circuits for a more sophisticated on-chip flow control. Here discrete circuits such as logic gates but also analogue approaches seem feasible. By combining the efforts of technological downscaling with model-based computer aided design we hope to enhance the potential of the LoC platform by the effects of integration and automation known from electronics.

## Supporting Information

S1 Fig Swelling Graph. Swelling behaviour of pure PNIPAAm and a co-polymerization of PNIPAAm with a 2.5% fraction of sodium acrylate over a series of ethanol concentrations (0wt% to 100.0wt%).
(PDF)

S1 File Control channel CFD simulation. Analysis of the control channel CFD simulation.
(PDF)

S2 Fig Control channel CFD simulation. CFD simulation in the control channel. Target value is the pressure within the hydrogel seat. (left)—Simulation without hydrogel particle. (right)—Simulation with swollen hydrogel particle.
(PDF)

S2 File Dynamic mechanical investigation. Analysis of the dynamic mechanical investigation.
(PDF)

S3 Fig Dynamic mechanical investigation. Dynamic mechanical behavior of the MIS-CVPT. Normalized amplitude of the flow rate and the exciting pressure over the frequency.
(PDF)

S3 File Data analysis and model fit. Explanation of the data analysis and model fit.
(PDF)

S4 Fig Data analysis and model fit. Exemplary plot of data points of the flow rate over pressure and ethanol concentration at 25°C with the according model fit.
(PDF)
