# Supplementary figures and images for: Integrated Microfluidic Membrane Transistor Utilizing Chemical Information for On-Chip Flow Control
Source: PLoS One. 2016 Aug 29;11(8):e0161024. doi: 10.1371/journal.pone.0161024 (PMC5003340; doi:10.1371/journal.pone.0161024)

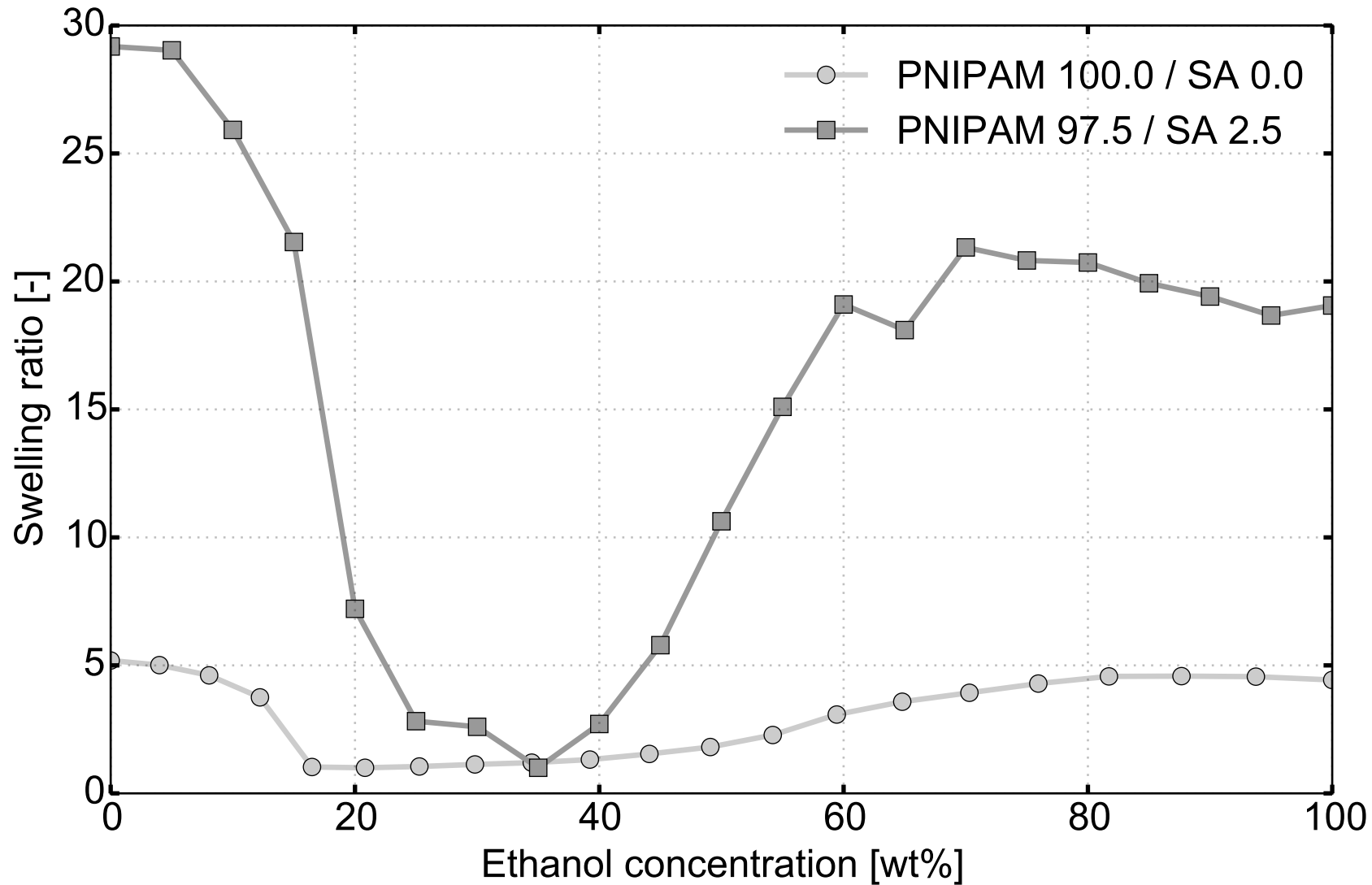

Supplement: S1 Fig — Swelling behaviour of pure PNIPAAm and a co-polymerization of PNIPAAm with a 2.5% fraction of sodium acrylate over a series of ethanol concentrations (0wt% to 100.0wt%). (PDF) [file pone.0161024.s001.pdf]

Pressure [mbar]

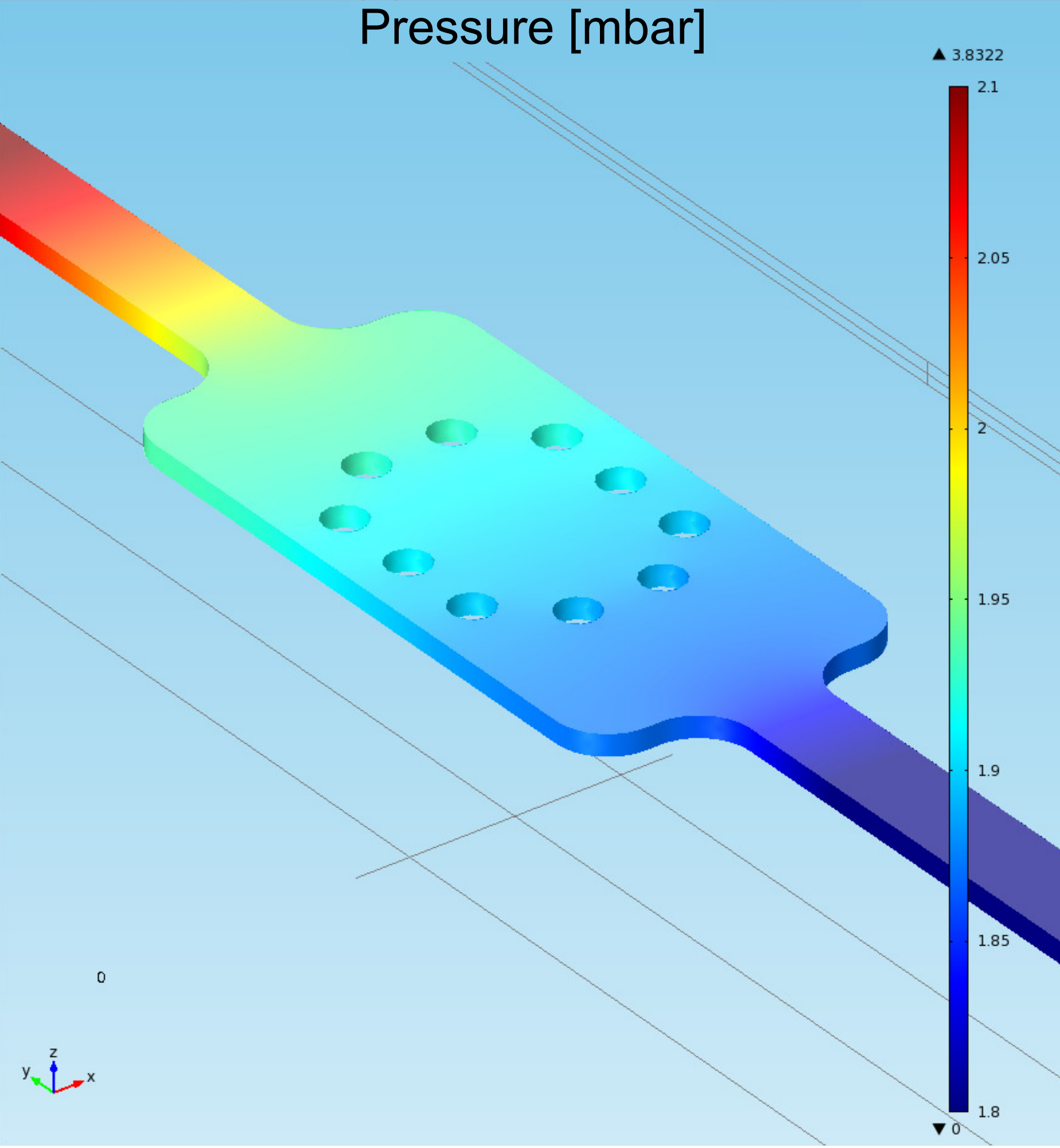

Pressure [mbar]

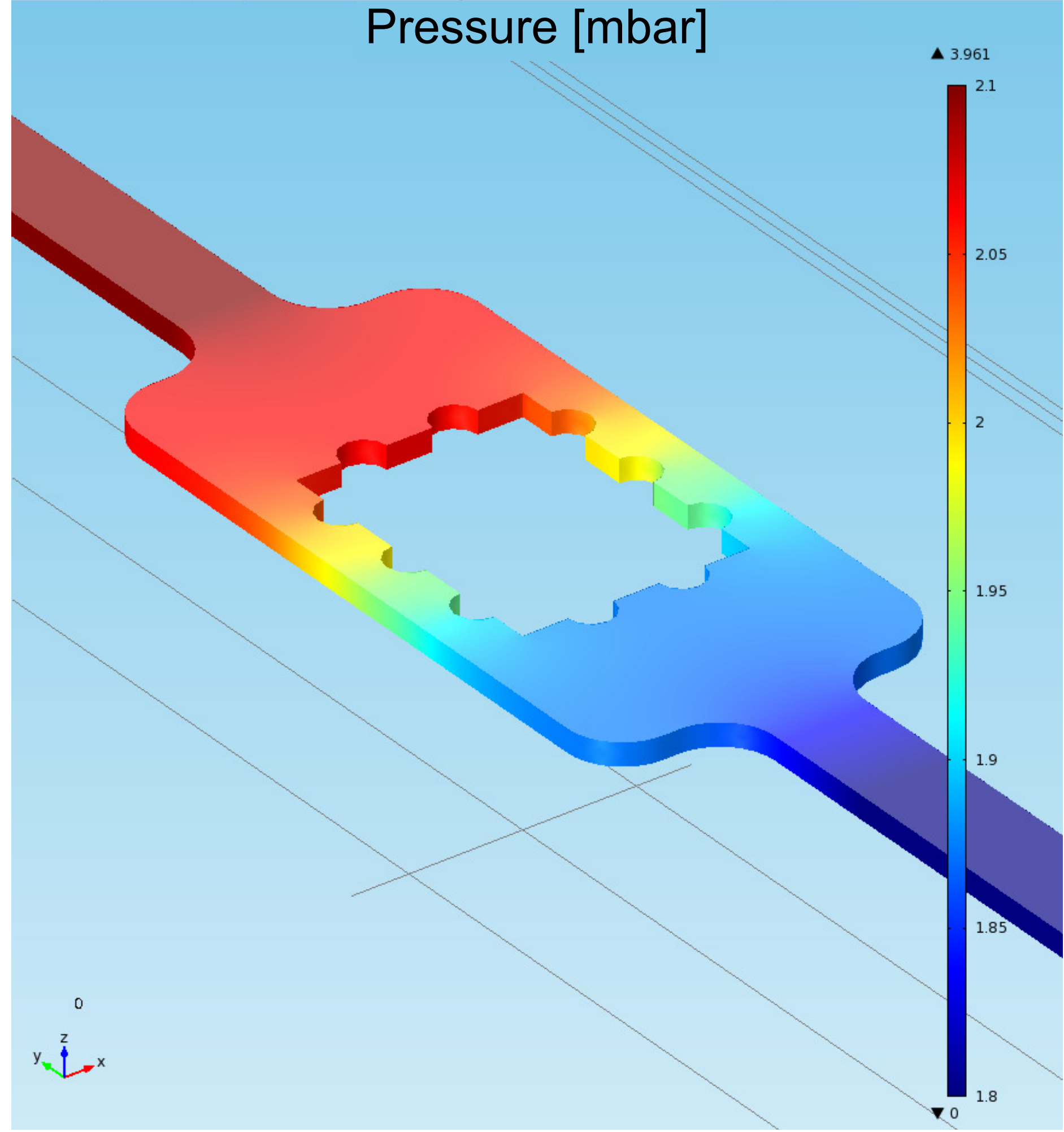

Supplement: S2 Fig — CFD simulation in the control channel. Target value is the pressure within the hydrogel seat. (left)—Simulation without hydrogel particle. (right)—Simulation with swollen hydrogel particle. (PDF) [file pone.0161024.s003.pdf]

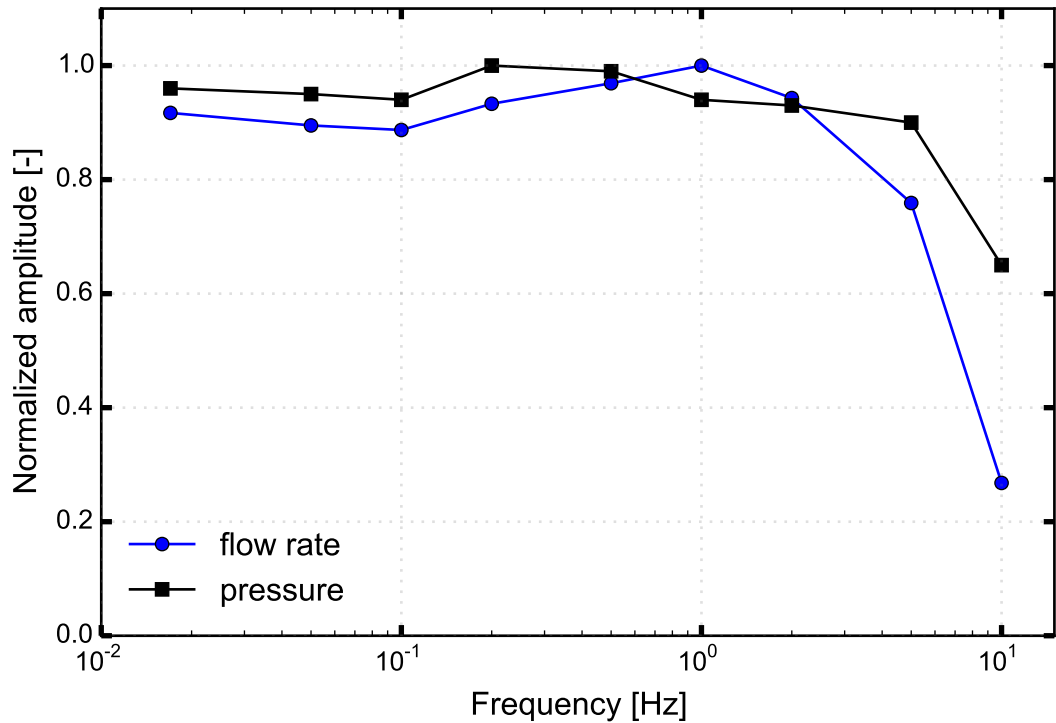

Supplement: S3 Fig — Dynamic mechanical behavior of the MIS-CVPT. Normalized amplitude of the flow rate and the exciting pressure over the frequency. (PDF) [file pone.0161024.s005.pdf]

$\vartheta = 25\text{ }^{\circ}\text{C}$

model —  
measurement x

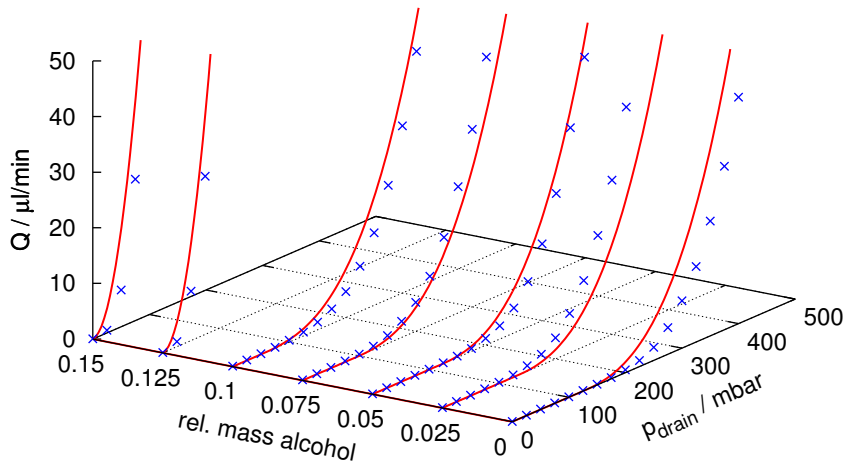

Supplement: S4 Fig — Exemplary plot of data points of the flow rate over pressure and ethanol concentration at 25°C with the according model fit. (PDF) [file pone.0161024.s007.pdf]
