# Supplementary material for: Integrated Microfluidic Membrane Transistor Utilizing Chemical Information for On-Chip Flow Control
Source: PLoS One. 2016 Aug 29;11(8):e0161024. doi: 10.1371/journal.pone.0161024 (PMC5003340; doi:10.1371/journal.pone.0161024)
Supplement: S1 File — Analysis of the control channel CFD simulation. (PDF) [file pone.0161024.s002.pdf]

Supplementary Material

S3: Control channel CFD simulation

A CFD simulation was performed to investigate the possible effect of the pressure in the control channel. COMSOL 4.2 was used for the CFD simulation. The simulation was in 3D for laminar flow physics and no-slip wall boundary condition. The laminar inflow into the inlet of the control channel was set to $15\,\mu\mathrm{L}\,\mathrm{min}^{-1}$, as in the experiment. The results of the CFD simulation can be found in Figure S3 Fig.

Over the experiments the volume of the gel structure varies in a wide range, altering the fluidic resistance of the control channel. Since the control channel was supplied with a constant flow rate to keep the gel homogeneously perfused to hold the environmental conditions constant, the pressure, which builds up in the channel, varies according to the channel's resistance. This counter pressure pushes against the membrane and influences to a certain degree the excitation pressure applied in the flow channel and therefore the measurements. A CFD simulation in COMSOL was employed to investigate to what extent this pressure influences the outcome of the experiments. Therefore two extreme cases for simulation were considered. The first case (Fig. S3 (left)) assumes a control channel with absent hydrogel and a second case assumes a fully swollen hydrogel, which blocks the seat completely. The region of interest is in both pictures horizontally in the middle of the seat structure. The maximum pressure drop in the simulation with no gel is at 3.83 mbar. The pressure at the region of interest lies at 1.92 mbar. The simulation with gel particle in place shows a maximum pressure of 3.96 mbar. The pressure at the hydrogel seat is at 1.97 mbar. The influence of the presence of the gel is generally small as the total pressure building up over the structure only slightly increases with the swelling of the particle. Therefore the fluidic resistance of the particle seat is negligible compared to the inlet and outlet channel adjacent. The pressure difference between absent gel and swollen state is 0.05 mbar. The applied pressure changes during the experiments were 25 mbar, which is 500 times higher than the counter pressure induced by the perfusion within the control channel. The simulation concludes that the pressure drop caused by the perfusion with a constant flow rate in order to keep the hydrogel under constant environmental conditions is negligible compared to the applied excitation pressure in the flow channel and therefore does not affect the measurements.
