# Supplementary material for: Integrated Microfluidic Membrane Transistor Utilizing Chemical Information for On-Chip Flow Control
Source: PLoS One. 2016 Aug 29;11(8):e0161024. doi: 10.1371/journal.pone.0161024 (PMC5003340; doi:10.1371/journal.pone.0161024)
Supplement: S2 File — Analysis of the dynamic mechanical investigation. (PDF) [file pone.0161024.s004.pdf]

Supplementary Material

S2: Dynamic mechanical investigation

In this study we determined the dynamic mechanical behavior of the device with the dimensions (1200 x 800) μm^2 by applying a sine pressure function at a specific operating point at a constant temperature and ethanol concentration. We chose the operating point temperature at 20 °C and ethanol concentration at 2.5 wt %. For excitation we used a sine pressure function with various periodic times. In the analysis we normalized the amplitude to the highest value accordingly of the exciting pressure and the resulting flow rate and plotted the graph over the frequency as seen in Fig. S2. The flow rate remains at the same level until a frequency of 2 Hz. For higher frequencies the amplitude drops, because the membrane cannot deflect to a maximum anymore and the system is unable to follow the exciting pressure due to damping by internal friction. Also the amplitude of the exciting pressure decreases after reaching a frequency of 5 Hz. The fact that the amplitude drop of the flow rate occurs at a lower frequency than the amplitude drop of the pressure proofs that the experiment was sufficient to determine the dynamic mechanical limit of the system.

However, since we are interested in the ability of the MIS-CVPT to respond to chemical input, we need to approximate the time limitation of the system in the chemical domain. Our dynamic chemical investigation in the main article shows, that the response time even of the fastest system lies in the min-range and therefore is still some magnitudes higher than the mechanical response. So we can conclude that the dynamic mechanical behavior is vastly sufficient for the application in the chemical domain.
