# Supplementary material for: Integrated Microfluidic Membrane Transistor Utilizing Chemical Information for On-Chip Flow Control
Source: PLoS One. 2016 Aug 29;11(8):e0161024. doi: 10.1371/journal.pone.0161024 (PMC5003340; doi:10.1371/journal.pone.0161024)
Supplement: S3 File — Explanation of the data analysis and model fit. (PDF) [file pone.0161024.s006.pdf]

Supplementary Material

S4: Data analysis and model fit

For the data fit, measurement data were arranged in the form of flow as a function of drain pressure, with control concentration and temperature as parameters. Control channel pressure and source pressure had been kept at zero in the measurements. From each of these data sets, first the threshold pressure $\mathrm{p}_0^* = 2\ \mathrm{p}_0$ at the drain side was extracted. To fit the slightly non-linear dependency of p_0^* on temperature and control concentration, a two-dimensional 2nd order polynomial, clipped at zero, was fit to the data - see Fig. 7. Then, a polynomial of order 4 was fitted to the data points with $\mathrm{p}_{drain} > p_0^*$. It turned out that a pure quadratic function in the shrunken cases and a cubic function in the swollen state best fits the measurement data. Also, the polynomial coefficients have no clear dependency on the parameters. Until more measurements and a more thorough investigation of the membrane and hydrogel mechanics will have been conducted, uniform values of these coefficients are assumed within the two different modes of operation. The quasi-static model expresses the hydrodynamic conductance G as a function of drain, source and control pressure:

$$\mathrm{p} = \frac{\mathrm{p}_{\mathrm{drain}} - \mathrm{p}_{\mathrm{source}}}{2} - \mathrm{p}_{\mathrm{c}} \tag{1}$$

$$\mathrm{G} = \begin{cases} 0 & \mathrm{p} \leq \mathrm{p}_0 \\ a_1(\mathrm{p} - \mathrm{p}_0) + a_2(\mathrm{p} - \mathrm{p}_0)^2 & \text{else} \end{cases} \tag{2}$$

Concentration c (relative mass) in the control channel and temperature ϑ determine the opening threshold pressure p_0, and the polynomial parameters a_1 and a_2 as follows:

$$\mathrm{p}_{\mathrm{x}} = \mathrm{p}_{\mathrm{x}0} + \mathrm{c}_1(\mathrm{c} - \mathrm{c}_0)^2 + \vartheta_1(\vartheta - \vartheta_0)^2 \tag{3}$$

with $\mathrm{p}_{x0} = 600\,\mathrm{mbar}$, $\mathrm{c}_0 = 0.02$, $\mathrm{c}_1 = 13\,000\,\mathrm{mbar}$, $\vartheta_0 = -15\,°\mathrm{C}$, $\vartheta_1 = -0.29\,\mathrm{mbar\,K}^{-2}$, $\mathrm{a}_{10} = 8\mathrm{e}^{-3}$, $\mathrm{a}_{20} = 2\mathrm{e}^{-6}$.

$$\mathrm{p}_0 = \begin{cases} 0 & \mathrm{p}_{\mathrm{x}} < 0 \\ \mathrm{p}_{\mathrm{x}} & \text{else} \end{cases} \tag{4}$$

$$\begin{aligned} \mathrm{a}_1 &= \mathrm{a}_{10} \cdot \mathrm{s}_1(\mathrm{p}_{\mathrm{x}}) \\ \mathrm{a}_2 &= \mathrm{a}_{20} \cdot \mathrm{s}_2(\mathrm{p}_{\mathrm{x}}) \end{aligned} \tag{5}$$

with

$$\begin{aligned} \mathrm{s}_1(\mathrm{p}_{\mathrm{x}}) &= \frac{1}{2}\left(1 - \tanh\left(\frac{\mathrm{p}_{\mathrm{x}} - 40\ \mathrm{mbar}}{2\ \mathrm{mbar}}\right)\right) \\ \mathrm{s}_2(\mathrm{p}_{\mathrm{x}}) &= \frac{1}{2}\left(1 + \tanh\left(\frac{\mathrm{p}_{\mathrm{x}} - 20\ \mathrm{mbar}}{2\ \mathrm{mbar}}\right)\right) \end{aligned} \tag{6}$$

Where the functions s_1 and s_2 have been introduced to switch the conductance from linear to quadratic behavior (see equation 2). Finally, flow in the controlled channel is modeled as:

$$\mathrm{q} = \mathrm{G} \cdot (\mathrm{p}_{\mathrm{drain}} - \mathrm{p}_{\mathrm{source}}). \tag{7}$$
